# Appendiceal carcinoid in a pediatric patient with Peutz-Jeghers syndrome: A case report and comprehensive literature review

**DOI:** 10.1097/MD.0000000000027389

**Published:** 2021-10-01

**Authors:** Zlatan Zvizdic, Emir Milisic, Nermina Ibisevic, Irmina Sefic Pasic, Semir Vranic

**Affiliations:** a Clinic of Pediatric Surgery, University Clinical Center Sarajevo, Sarajevo, Bosnia and Herzegovina; b Department of Pathology, University Clinical Center Sarajevo, Sarajevo, Bosnia and Herzegovina; c Department of Radiology, University Clinical Center Sarajevo, Sarajevo, Bosnia and Herzegovina; d College of Medicine, QU Health, Qatar University, Doha, Qatar; e Biomedical and Pharmaceutical Research Unit, QU Health, Qatar University, Doha, Qatar.

**Keywords:** appendix, carcinoid, children, hamartomas, intussusception, Peutz-Jeghers syndrome

## Abstract

**Rationale:**

Peutz-Jeghers syndrome (PJS), a rare autosomal dominant disorder, is characterized by mucocutaneous pigmentations, hamartomatous polyps in the gastrointestinal tract, and a high risk of developing various malignancies. To the best of our knowledge, only 1 case of appendiceal carcinoid associated with PJS has been previously reported in the pediatric population.

**Patient concerns:**

We report a 7-year-old girl who was admitted for severe, intermittent abdominal pain and cramps, nausea, and vomiting. Multiple brown melanotic macules on the lips, buccal mucosa, and the tongue were noted.

**Diagnosis:**

A plain abdominal X-ray in a standing position revealed dilated intestinal loops with multiple air-fluid levels. A computed tomography scan of the abdomen showing a “coffee bean” appearance of the jejunal loop with a transition point to the duodenal loop. Axial-contrast-enhanced computed tomography scan of the abdomen showing dilated jejunum loops, filled with fluid with the swirled appearance of mesentery typical for volvulus. The diagnosis of PJS was based on clinical findings along with the histopathologic confirmation of the hamartomatous polyps.

**Interventions:**

An emergency laparotomy was performed, revealing a jejunojejunal intussusception starting 40 cm from the duodenojejunal flexure. Jejunotomy revealed that a lead-point intussusception was a necrotic hamartomatous polyp. After resecting the involved jejunal necrotic segment, including the polyp, end-to-end jejuno-jejunal anastomosis was performed. Further exploration revealed the presence of a jejunal mass 80 cm from the duodenojejunal flexure identified as another hamartomatous pedunculated polyp. The polyp was resected, and the enterotomy was then closed transversely. The grossly normal appendix was also removed.

**Outcomes:**

Clinical findings along with the histopathologically confirmed hamartomatous polyps were consistent with PJS. An appendiceal carcinoid (well-differentiated neuroendocrine tumor, European Neuroendocrine Tumor Society stage pT2) was incidentally detected during histological examination of the appendix. The patient and parents were counseled accordingly, focusing on active surveillance and control of symptoms. Two additional hamartomatous polyps (gastric and jejunal) were detected endoscopically and resected in the fourth postoperative week. A regular, 1-year follow-up and surveillance revealed no complications or recurrences.

**Lessons:**

Unusual neoplasms can occasionally be encountered in well-defined syndromes such as PJS. Therefore, active follow-up and surveillance are mandatory for all patients with PJS.

## 1 Introduction

The Peutz-Jeghers syndrome (PJS) is an autosomal dominant disease (OMIM#175200) caused by a germline mutation of the serine/threonine kinase 11 (*STK11*) gene on chromosome 19p13.3.^[1,2]^ PJS is characterized by mucocutaneous pigmentations, hamartomatous polyps in the gastrointestinal (GI) tract, and a significantly increased risk of various cancers, particularly in females.^[1,2]^ The incidence of PJS is estimated to be between 1 in 50,000 to 1 in 200,000 live births.^[3,4]^

The diagnosis of PJS is made clinically when at least 2 of the following 3 diagnostic criteria are met: mucocutaneous lentiginosis, a family history of PJS, and at least 2 hamartomatous polyps of the GI tract.^[3]^ Frequent complications in patients with PJS include bleeding, obstruction, and intussusception.^[4]^

Patients with PJS have an increased risk of various cancers and shortened life expectancy.^[5]^ Most cancers originate from the GI tract, followed by breast, ovarian, cervical, lung, pancreatic, uterine, and testicular tumors.^[3,5]^

We herein present a case of a 7-year-old girl with appendiceal carcinoid as an incidental finding on laparotomy for jejunojejunal intussusception. Intussusception was caused by jejunal hamartomatous polyps, which were parts of the PJS spectrum. To the best of our knowledge, only 1 case of appendiceal carcinoid associated with PJS has been reported in the pediatric population so far.^[6]^ Our patient is probably the youngest case reported so far.

## 2 Case report

A 7-year-old girl was admitted to our department for severe, intermittent abdominal pain and cramps, nausea, and vomiting. She appeared lethargic with generalized weakness but conscious. These symptoms were gradually intensified within 24 hours of the onset of pain.

**Figure 1:**
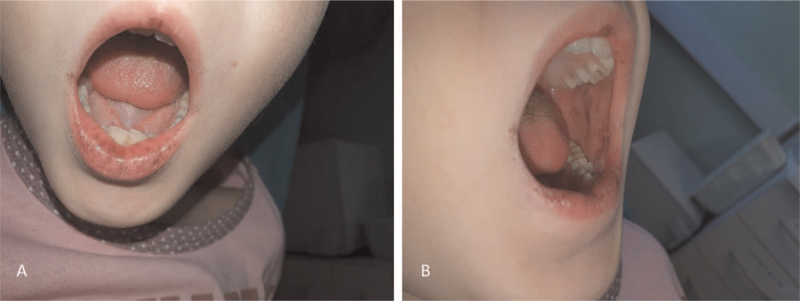
A–B. The dark pigmented spots (melanocytic macules) affecting the lips, buccal mucosa, and the tongue in a 7-year-old girl.

**Figure 2:**
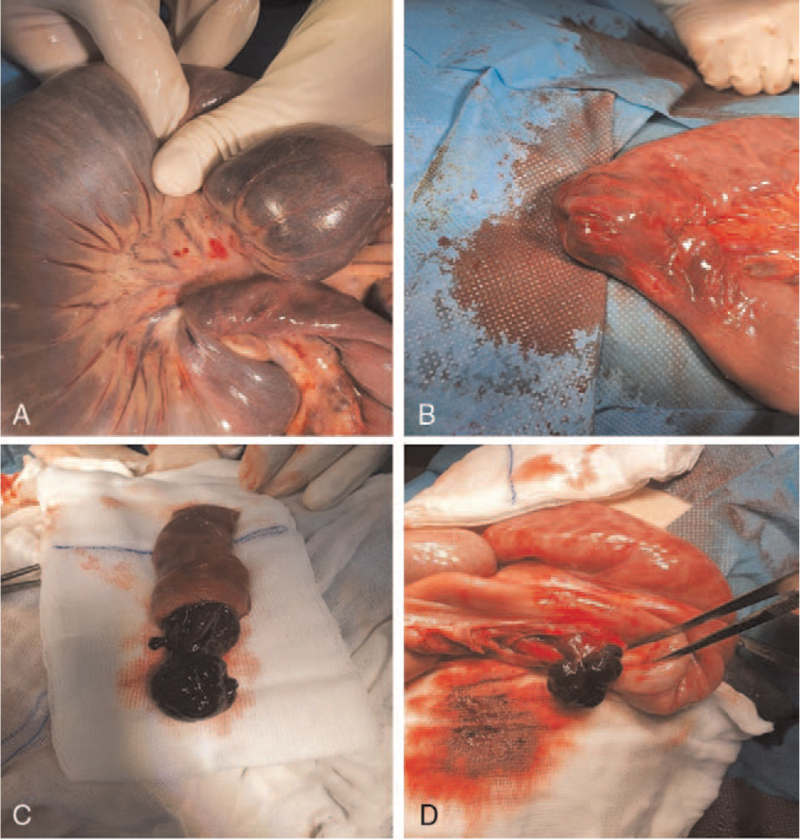
A–D. Reformatted coronal CT scan of the abdomen showing “coffee bean” appearance of the jejunal loop (red arrow) with transition point to duodenal loop (pointed red arrow) (A and C). Axial-contrast-enhanced CT scan of the abdomen showing dilated jejunum loops, filled with fluid (red arrows) with the swirled appearance of mesentery typical for volvulus (pointed red arrow) (B). Reformatted sagittal CT scan showing dilated jejunal loop with mild postcontrast enhancing (red arrow) and swirled mesentery (pointed red arrow) (D). CT = computed tomography.

Physical examination was remarkable for epigastric and periumbilical tenderness and mild abdominal distention. In addition, multiple brown melanotic macules on the lips, buccal mucosa, and the tongue were noted (Fig. 1A and B). A plain abdominal X-ray in a standing position revealed dilated intestinal loops with multiple air-fluid levels. The abdominal ultrasonography findings suggested small bowel intussusception, but the presumed pathologic lead point for the intussusception was not found. Reformatted coronal computed tomography (CT) scan of the abdomen showing a “coffee bean” appearance of the jejunal loop with a transition point to the duodenal loop (Fig. 2A and C). Axial-contrast-enhanced CT scan of the abdomen showing dilated jejunum loops, filled with fluid with the swirled appearance of mesentery typical for volvulus (Fig. 2B). Reformatted sagittal CT scan showing dilated jejunal loop with mild postcontrast enhancing and swirled mesentery (Fig. 2D).

**Figure 3:**
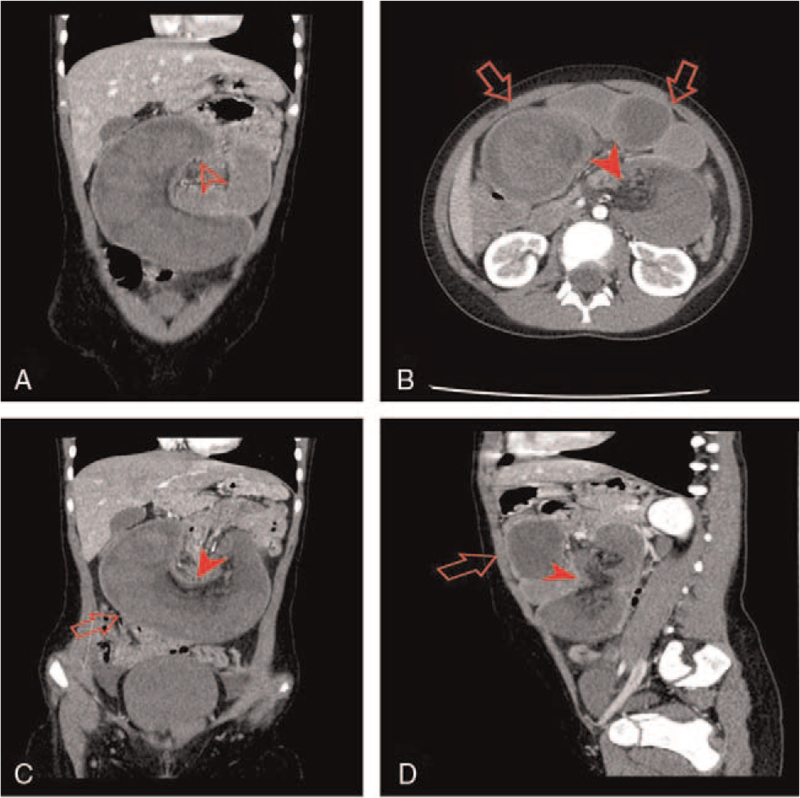
A-D. Intraoperative view of the jejunojejunal intussusception (A); Hamartomatous polyp was the leading point of intussusception (B); The resected jejunal segment, including the polyp (C); Another hamartomatous pedunculated polyp in the lower jejunum (D).

**Figure 4:**
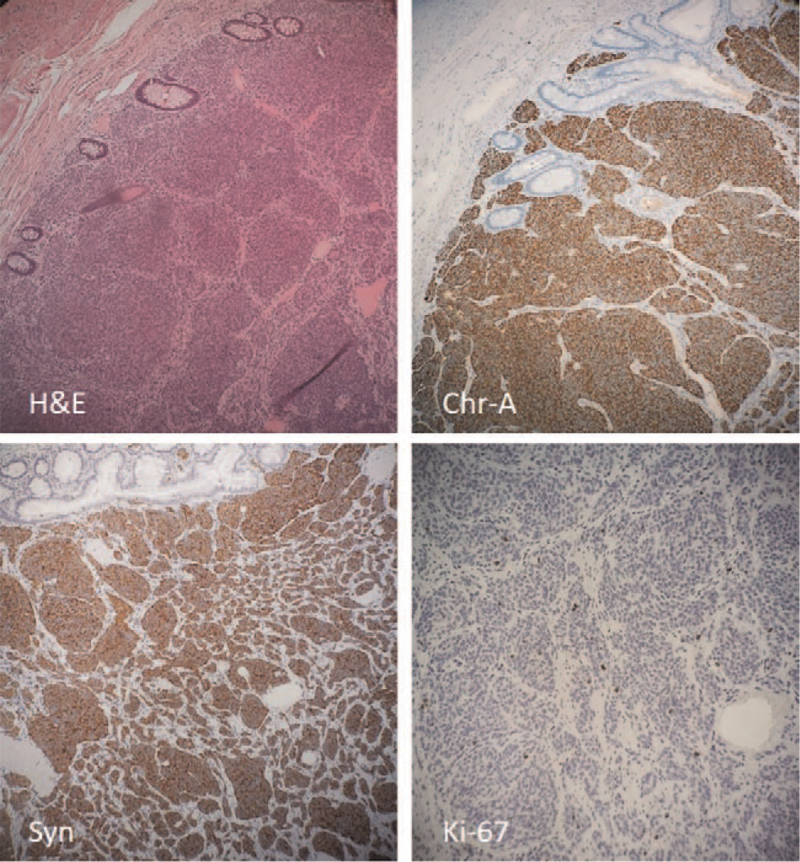
A-D. Hematoxylin and eosin (H&E) slide revealing a well-differentiated neuroendocrine tumor (carcinoid, G1) infiltrating the appendiceal wall; neoplastic cells were positive for neuroendocrine markers: Chromogranin-A (Chr-A) and Synaptophysin (Syn); the proliferation rate of the neoplastic cells was low (<1%) as measured by Ki-67 (MIB-1 antibody).

After urgent resuscitation and antibiotics, the patient was taken for emergency laparotomy. This revealed a jejunojejunal intussusception starting 40 cm from the duodenojejunal flexure (Fig. 3A). The gently manual reduction was made, but jejunal segmental necrosis was found. Jejunotomy revealed that a lead-point intussusception was a necrotic hamartomatous polyp (Fig. 3B). After resecting the involved jejunal necrotic segment (approximately 10 cm long), including the polyp (Fig. 3C), end-to-end jejuno-jejunal anastomosis was performed. Further exploration revealed the presence of a jejunal mass 80 cm from the duodenojejunal flexure identified as another hamartomatous pedunculated polyp (Fig. 3D). The polyp was resected, and the enterotomy was then closed transversely. The macroscopically normal appendix was also removed. The postoperative course was uneventful. Histologic findings confirmed the hamartomatous polyps. However, the histopathological examination of the appendix revealed an 8 × 6 mm large well-differentiated neuroendocrine tumor (carcinoid); the tumor cells infiltrated submucosa, muscularis propria, with minimal infiltration of the subserosa/mesoappendix (European Neuroendocrine Tumor Society stage pT2)^[7]^ (Fig. 4A). The tumor cells showed low mitotic activity (<2 mitoses/2 mm^2^) and a low Ki-67 index (<1%) (Fig. 4D). The tumor cells were also diffusely positive for neuroendocrine markers Chromogranin-A (Fig. 4B) and Synaptophysin (Fig. 4C). These findings were consistent with a low-grade (G1) neuroendocrine tumor (carcinoid).

The patient had a negative family history of PJS or other hereditary cancers, and both parents were healthy. Routine *STK11* genetic testing was not available. Given that the clinical criteria for the diagnosis of PJS were fulfilled,^[2,3,8,9]^ the counseling was provided,^[2]^ focusing on active surveillance and control of symptoms.

In the fourth postoperative week, upper GI endoscopy and colonoscopy were done, revealing 2 additional hamartomatous polyps, first in the gastric fundus and the second in the jejunum. Endoscopic polypectomies were done, and hamartomatous polyps were histopathologically confirmed. The child is under regular follow-up and surveillance, and no recurrences or further complications have been found within a 1-year follow-up.

## 3 Discussion

This case report describes a rare association between the appendiceal carcinoid (well-differentiated neuroendocrine tumor) and PJS in a prepubertal girl. The patient had no family history of PJS, indicating a possible de novo *STK11* mutation, which is present in ∼45% of affected individuals with a negative family history of PJS.^[10]^ It is also well-documented that ∼50% of the patients with PJS have to undergo surgery by age 18 because of polyps-related complications, including abdominal pain, rectal bleeding, anemia, bowel obstruction, or intussusception.^[11]^ Intussusception caused by intraluminal polyps in PJS is a rare cause of intestinal obstruction. It can potentially cause life-threatening complications. Unlike a classic clinical triad of intussusception (colicky/intermittent abdominal pain, vomiting, and red “currant jelly” stool), our patient did not have rectal bleeding. Since ultrasonography has a low sensitivity for certain causes of intussusception, such as intraluminal polyps,^[12]^ the cause of intussusception in our case was explored by surgery. However, pre-operative CT in our patient showed evidence of small-bowel obstruction with focal intussusception.

Although the mechanism of carcinogenesis remains debatable, the patients with PJS have a 93% life-time-risk for various cancers,^[13]^ predominantly of GI origins.^[5]^ Utsunomiya et al^[14]^ reported that benign complications of the polyps, such as bleeding and intussusception, predominate in the first 3 decades of life. In contrast, malignant complications become more common after that. The clinical presentation of our patient with polyp-induced intussusception confirmed this observation.

The causal relationship between PJS and appendiceal carcinoid remains unclear. Our comprehensive literature survey (PubMed/MEDLINE, Scopus, and Web of Science Core Collection) revealed only 1 case of appendiceal carcinoid in patients with PJS.^[6]^ However, other GI neuroendocrine neoplasms (carcinoid of the rectum and neuroendocrine carcinoma of the duodenum) have been reported in adult patients with PJS^[15,16]^ (Table 1). Also, other appendiceal malignancies (adenocarcinoma) have been found in patients with PJS.^[17–20]^ In addition, Mojsilovic et al^[21]^ reported a 3-generation family with PJS followed over 37 years. Among others, the authors found endocrine hyperplasia of chromogranin and serotonin-positive cells while some family members had clinically carcinoid-like symptoms^[21]^ (Table 1). The link between the PJS and neuroendocrine neoplasms is not clear. However, these tumors have been closely linked to several related cancer syndromes such as neurofibromatosis type 1 and tuberous sclerosis complex.^[22]^ Lodish et al^[22]^ provided the hypothesis that the deregulation of the mammalian target of rapamycin pathway might be an underlying mechanism for specific tumors in these related syndromes.

**Table 1:** Overview of the studies that reported neuroendocrine neoplasms of the gastrointestinal tract in patients with Peutz-Jeghers syndrome.

Study (year) (reference)	Age (years)	Gender	Diagnosis	Anatomic location
Mojsilovic et al (2015)^[21]^	21 (initially diagnosed with PJS at the age of 8)	Male	Neuroendocrine hyperplasia (Chr-A and serotonin positive cells)	Jejunum and ileum
Hofmann et al (2014)^[6]^	21	Male	Carcinoid	Appendix
Chen et al (2012)^[16]^	42	Male	Neuroendocrine carcinoma	Duodenum
Wada et al (1998)^[15]^	8	Male	Carcinoid	Rectum

Chr-A = Chromogranin-A, PJS = Peutz-Jeghers syndrome.

The most common appendiceal tumors are mucinous neoplasms (e.g., low-grade appendiceal mucinous neoplasm and mucinous carcinomas), adenomas, serrated polyps, goblet cell tumors, neuroendocrine tumors, and colonic type carcinomas.^[23]^ Among these tumors, appendiceal carcinoids, neoplasms derived from the subepithelial neuroendocrine cells of the appendix, are the most frequent tumors, accounting for up to 60% of all appendiceal tumors.^[24]^ Although the appendix is the most common site for carcinoids in children,^[25,26]^ these tumors are very rare, with the reported frequency of ∼0.2% to 0.4% of all pediatric appendectomies.^[27,28]^ The clinical presentation of symptomatic cases is similar to that of acute appendicitis. However, appendiceal carcinoids are usually asymptomatic and are detected incidentally during surgery,^[29]^ as recorded in our case. In addition, appendiceal carcinoids are associated with the most favorable survival rates compared with other neuroendocrine tumors.^[30]^ The management algorithm for appendiceal carcinoids developed 3 decades ago has not changed significantly to date.^[31,32]^ However, because of the rarity of carcinoid tumors in children, management guidelines have been challenging to generate.^[33]^ Although extended resection and right colectomies are currently recommended for tumors >2 cm in adults, this treatment modality is inconsistent in the pediatric population due to reports that children with tumors ≥2 remained disease-free for 10 years after appendectomy alone.^[34,35]^ For this reason, there are recommendations for less aggressive management of appendiceal carcinoids in children.^[34]^ Given the tumor size in our patient (<1 cm), we also opted for a less aggressive surgical approach and active surveillance of the PJS patient.^[2]^

In conclusion, PJS is a rare inherited syndrome associated with various non-neoplastic and neoplastic complications, both of which require thorough surveillance and regular follow-up. Furthermore, GI carcinoids, including the appendiceal reported in our study, have rarely been described in PJC patients, which merits further research.

## Author contributions

**Conceptualization:** Zlatan Zvizdic, Semir Vranic.

**Data curation:** Zlatan Zvizdic, Emir Milisic, Nermina Ibisevic, Irmina Sefic Pasic, Semir Vranic.

**Formal analysis:** Zlatan Zvizdic, Semir Vranic.

**Investigation:** Zlatan Zvizdic, Emir Milisic, Nermina Ibisevic, Irmina Sefic Pasic.

**Writing – original draft:** Zlatan Zvizdic, Semir Vranic.

**Writing – review & editing:** Zlatan Zvizdic, Semir Vranic.
